# Multidecadal poleward shift of the southern boundary of the Antarctic Circumpolar Current off East Antarctica

**DOI:** 10.1126/sciadv.abf8755

**Published:** 2021-06-11

**Authors:** Kaihe Yamazaki, Shigeru Aoki, Katsuro Katsumata, Daisuke Hirano, Yoshihiro Nakayama

**Affiliations:** 1 Graduate School of Environmental Science, Hokkaido University, Sapporo, Japan.; 2 Institute of Low Temperature Science, Hokkaido University, Sapporo, Japan.; 3 Japan Agency for Marine-Earth Science and Technology, Yokosuka, Japan.; 4 Arctic Research Center, Hokkaido University, Sapporo, Japan.

## Abstract

The southern boundary (SB) of the Antarctic Circumpolar Current, the southernmost extent of the upper overturning circulation, regulates the Antarctic thermal conditions. The SB’s behavior remains unconstrained because it does not have a clear surface signature. Revisited hydrographic data from off East Antarctica indicate full-depth warming from 1996 to 2019, concurrent with an extensive poleward shift of the SB subsurface isotherms (>50 km), which is most prominent at 120°E off the Sabrina Coast. The SB shift is attributable to enhanced upper overturning circulation and a depth-independent frontal shift, generally accounting for 30 and 70%, respectively. Thirty years of oceanographic data corroborate the overall and localized poleward shifts that are likely controlled by continental slope topography. Numerical experiments successfully reproduce this locality and demonstrate its sensitivity to mesoscale processes and wind forcing. The poleward SB shift under intensified westerlies potentially induces multidecadal warming of Antarctic shelf water.

## Teaser

Poleward expansion of the Antarctic Circumpolar Current warms the Antarctic continental margin.

## INTRODUCTION

In recent decades, the Southern Ocean has undergone widespread but spatially heterogeneous warming. In the northern part of the Antarctic Circumpolar Current (ACC; 45° to 55°S), the temperature of the upper 1000 m has increased rapidly (0.1° to 0.2°C per decade), partly due to enhanced surface heat uptake (*1*). In contrast, multidecadal warming in the southern part of the ACC (55° to 65°S, subpolar zone) is confined to a deeper layer (below 200 m) and is relatively gradual (~0.1°C per decade) (*1*–*5*). Upwelling of subsurface cold water (<0°C), reinforced by intensified westerlies, has cooled the subpolar ocean surface (*6*). Deep warm salty water (below 200 m, temperature > 0°C, salinity > 34.85 g kg^−1^), known as Circumpolar Deep Water (CDW), compensates volumetrically for the near-surface divergence, and subsequent enhancement of poleward CDW upwelling may lead to warming below 200 m. CDW primarily supplies heat to the Antarctic continental shelves. Thus, multidecadal warming of the subpolar CDW layer has enormous implications for Antarctic ice shelves (*7*), global overturning circulation (*8*), and marine ecosystems (*9*).

Poleward CDW upwelling constitutes the upper and lower cells of the global overturning circulation (*10*). The poleward flank of the upper meridional overturning circulation has been identified as the southernmost extent of the oxygen-depleted layer, which dynamically corresponds to the southern boundary (SB) of the ACC (*10*). The SB is represented by the poleward limit of the 1.5°C isotherm in Upper CDW (*11*, *12*). In the upper overturning circulation, baroclinic (with depth-dependent structure) eddies transport CDW poleward, as the potential energy imposed by the westerlies is released through baroclinic instability (*7*, *10*). Given that the lagged correlation between eddy kinetic energy and surface winds (*13*, *14*) and isopycnal temperature increases (*15*), we expect that the multidecadal warming of the CDW layer is associated with enhanced eddy fluxes in response to wind intensification. Despite this dynamic background, the role of eddies in the multidecadal trend of wind-driven upper overturning circulation is not evident. CDW upwelling has been hypothesized to react to wind changes more slowly than the immediate response of surface water (*16*), whereas the contributions of eddy fluxes to multidecadal warming have not been constrained by previous studies that used both idealized ACC simulations and coupled climate models (*17*). In addition to eddy transport, the poleward displacement of the ACC fronts also results in warming of the CDW layer. The zonally averaged ACC position has not shifted decadally (1992–2013) (*7*) under the intensified westerlies from 1990 onward (*18*). However, this stability of the ACC does not preclude any regional trends (*19*) because the bathymetry globally steers zonal asymmetry of the ACC through its barotropic (depth-independent) nature (*20*, *21*).

Historical temperature records from the Southern Ocean have indicated significant warming of CDW off East Antarctica over the past several decades (*3*, *4*). Hydrography of the eastern Indian sector of the Southern Ocean (or the Australian-Antarctic Basin; 80° to 150°E) along 62°S, corresponding to the southern part of the ACC, has revealed a patchy pattern of poleward CDW migration from 1995 to 2012, which is concurrent with warming throughout the entire water column by 0.1° to 0.4°C along pressure surfaces (*22*). Satellite altimetry has indicated that the Polar and Southern ACC Fronts have shifted further poleward (by 60 to 120 km) in the eastern Indian sector than in the other sectors from 1993 to 2010 (*23*). Observed sea-level rise that significantly exceeds the global mean trend (1992–2011) (*24*) supports this rapid poleward shift of ACC fronts in the eastern Indian sector. The latest reanalysis of a 25-year record along the hydrographic section at 140°E has shown that multidecadal warming of CDW around the Southern ACC Front (61° to 55°S) dominates the interannual variability of 0.04 ± 0.01°C per decade (where ± indicates one SD) (*2*).

Nevertheless, little is currently known about the multidecadal trend of the SB. A previous study documented that the regional sea-level rise of 3.4 ± 0.48 cm from 1996 to 2008 in the western Indian sector (30° to 80°E) is equivalent to a poleward SB shift of 100 to 200 km (*25*). However, the meridional location of the SB derived from hydrographic data often differs by more than 100 km from the location derived from altimetry (*25*). The effects of global sea-level rise and increased freshwater discharges from land ice account for a substantial portion of the altimetric change (*24*). Regarding the physical configuration of the SB, which does not necessarily follow a geostrophic jet detectable by a peak in the sea surface height gradient (*12*), the use of satellite altimetry alone is insufficient to determine its variability. Therefore, we analyze the ocean interior to examine the connection between the SB and the multidecadal warming in the subpolar zone.

**Fig. 1:**
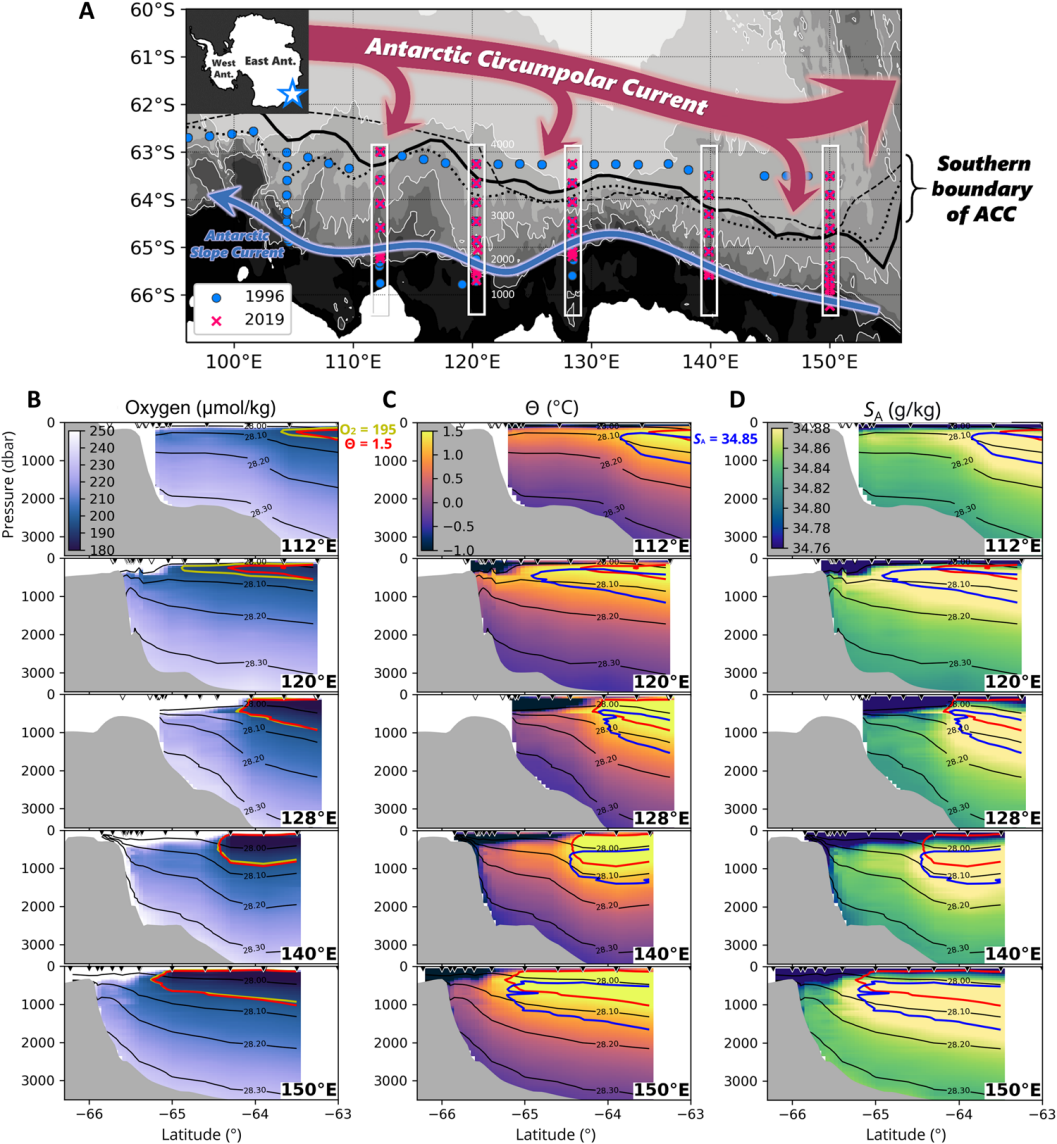
Revisited hydrography off East Antarctica. (**A**) Hydrographic arrays along the continental margin of the Australian-Antarctic Basin. CTD stations from the R/V *Aurora-Australis* 1996 cruise are blue dots, and CTD stations from the R/V *Kaiyo-maru* 2019 cruise are pink crosses. White boxes highlight the five revisited CTD sections. Background color indicates bathymetry (isobaths at 1000-m intervals). Black curves denote the climatological position of the SB [solid, historical dataset from this study; broken, Orsi *et al.* (*11*); dotted, Shimada *et al.* (*35*)]. Five sections of the 2019 measurements of (**B**) dissolved oxygen, (**C**) Conservative Temperature, and (**D**) Absolute Salinity. Isopycnal contours (neutral density) are shown in black (from 28.0 to 28.3 kg m^−3^ at 0.1 kg m^−3^ intervals). Yellow, red, and blue curves are the 195 μmol/kg oxygen, 1.5°C isotherm (represents Upper CDW), and 34.89 g kg^−1^ isohaline (represents Lower CDW), respectively. Black/white triangles at the top of each graph indicate the location of the CTD stations for the 2019 and 1996 observations.

Off East Antarctica (including eastern/western Indian sectors), the SB is located along the center of subpolar gyres and is meridionally bounded by the offshore ACC and the shoreward Antarctic Slope Current (*12*, *26*). Subpolar gyres are clockwise circulations that are typical along the continental margin near the Antarctic Divergence. At the eastern flank of the clockwise circulation, onshore CDW intrusion is maintained by southward flows that branch from the ACC (Fig. 1A) (*12*, *26*–*29*). Motivated by the ongoing mass loss of the East Antarctic Ice Sheet and its potential impact on the global sea-level rise (*30*), we delineate multidecadal changes in the SB in the eastern Indian sector. Results from hydrographic observations conducted in 2019 during the austral summer (Fig. 1A) are presented. This cruise revisited hydrographic sections surveyed in the summer of 1996 (*31*). Comparisons between the 1996 and 2019 measurements highlight a poleward shift of the SB in the eastern Indian sector and suggest possible mechanisms of observed multidecadal warming in the subpolar zone. Thirty years of oceanographic records from off East Antarctica support findings obtained from the 1996 and 2019 datasets. Furthermore, we conducted numerical experiments to investigate the possible factors controlling the changes in the SB.

## RESULTS

### Water column property trends from 1996 to 2019

The revisited meridional sections are located over the continental slope between the 1000- to 4000-m isobaths, with zonal intervals of 10° (Fig. 1A). The temperature maximum layer (represented by 1.5°C) presents around the 28.0 kg m^−3^ neutral density γ_n_, which characterizes the upper meridional overturning (*10*). The salinity maximum layer (represented by 34.89 g kg^−1^ Absolute Salinity) presents around the 28.1 kg m^−3^ isopycnal, which characterizes the lower meridional overturning (*10*). The abyssal oxygen-rich, cold, fresh water is Antarctic Bottom Water (AABW), which is present below the 28.2 kg m^−3^ isopycnal (defined by the extent of the salinity maximum layer). The 1.5°C isotherm observed in 2019 agrees with the 195 μmol/kg dissolved oxygen contour, where the oxygen-depleted layer abruptly attenuates poleward (Fig. 1B). The oxygen value at the southernmost extent of the 1.5°C isotherm coincides with the original description by Orsi *et al.* (*11*) and the 1996 measurements (*31*), underpinning the oxygen invariance along the isotherm required for a proxy of the SB. Thus, we define the SB as the poleward limit of the 1.5°C isotherm for the following analyses.

**Fig. 2:**
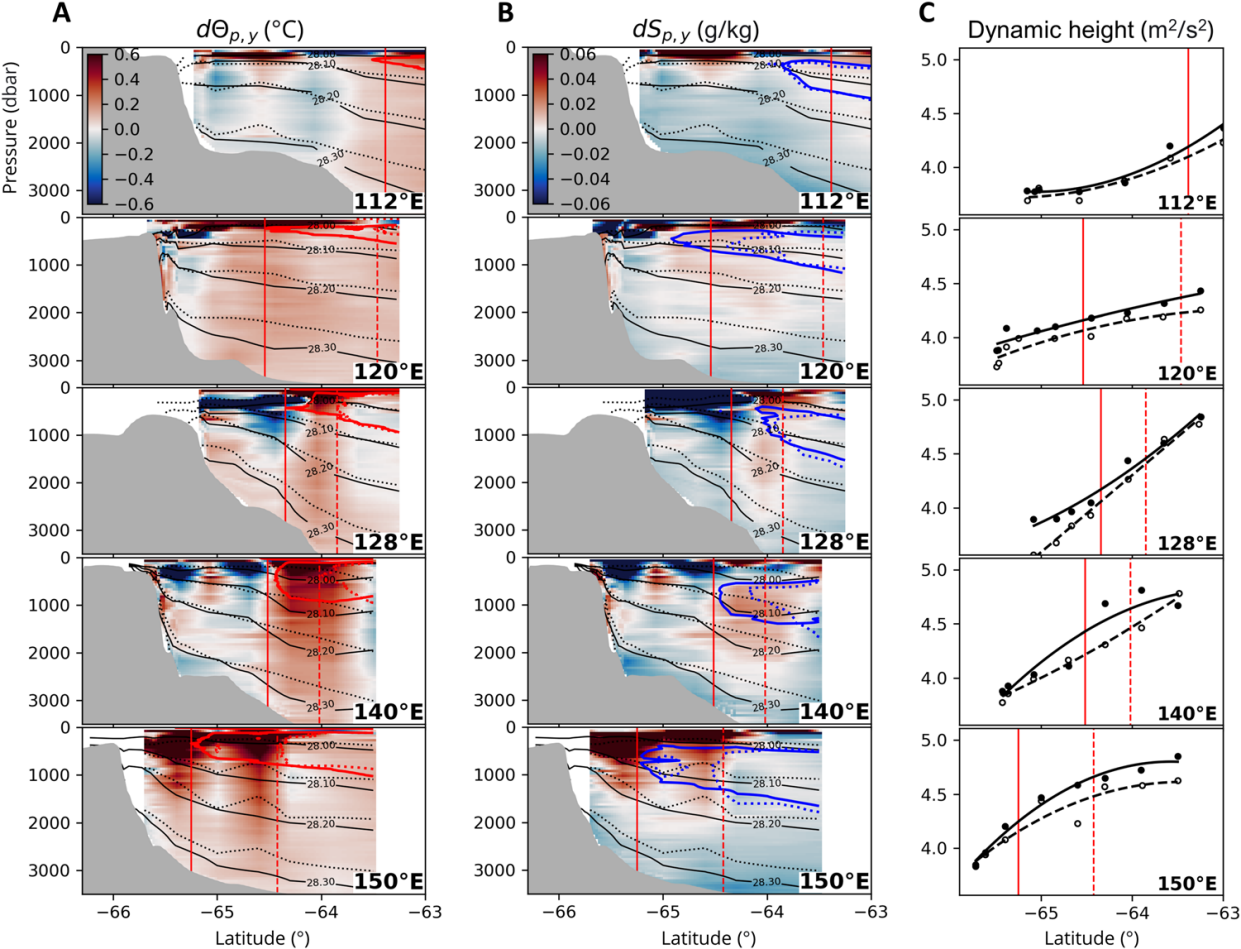
Isobaric trends from 1996 to 2019. Differences between 1996 and 2019 in (**A**) Conservative Temperature and (**B**) Absolute Salinity at the latitude-pressure coordinates. The isotherms, isohalines, and isopycnals are the same as in Fig. 1. The dotted lines denote those from 1996. The red vertical line is the position of the SB determined by the poleward limit of the 1.5°C. (**C**) Dynamic height between 300 and 1800 dbar. Dots are the values at each station, which are least-squares fitted by quartic polynomials. Black (white) dots and solid (dashed) profiles are from 2019 (1996).

A poleward shift of the SB by 50 to 120 km is observed in the five meridional sections between the 1996 and 2019 measurements, with an isobaric temperature increase in the upper 1500 dbar of more than 0.1°C (Fig. 2A and Table 1). The SB shift is greater than the meridional sampling intervals (<0.5° in latitude; indicated by triangles in Fig. 1, B to D) except 112°E, where the SB is absent in 1996. This temperature change is comparable to the reported warming of CDW further north (*2*, *3*, *22*). The poleward shift of the SB is most pronounced at 120°E off the Sabrina Coast (120 km). This displacement is accompanied by a deepening of the isopycnals throughout the entire water column by 100 to 400 dbar, with a density decrease that exceeds 0.02 kg m^−3^. Freshening prevails throughout most of the water column (Fig. 2B), likely associated with increased glacial water discharge (*24*) and changes in the properties of AABW (*22*, *32*, *33*). In contrast to the widespread freshening, a poleward migration of the salinity maximum layer (blue contour) appears with salinification in the CDW layer above 1500 dbar in the vicinity of the SB (see *dS*_A_ in Table 1), and the salinification exceeding 0.06 g kg ^−1^ spreads up to the surface further south of the SB at 112°, 120°, and 150°E. Although the freshening drives volume reduction of AABW denser than 28.2 kg m^−3^ (*24*, *32*), the salinity change is not the cause of isopycnal deepening because temperature overwhelms salinity in the steric changes below 1500 m by a factor of 6 to 8 (based on Table 1). The temperature-driven volume reduction of AABW is previously reported and attributed to isotherm heaving (*33*). We interpret the full-depth isopycnal deepening near the SB as being due to the poleward shift of the barotropic ACC fronts, which reasonably explains the isotherm heaving.

**Table 1:** Water column changes associated with the poleward SB shift. The poleward shift of the SB by the 1.5°C isotherm and the water property changes averaged over the latitudinal ranges of the 1996 and 2019 positions of the SB (with SD) for the five meridional sections. Isobaric changes above/below 1500 dbar are shown separately for Conservative Temperature, Absolute Salinity, and neutral density. In addition to the total isopycnal change, the frontal shift and the residual (water transformation) terms are presented for Upper CDW (28.0 to 28.1 kg m^−3^), Lower CDW (28.1 to 28.2 kg m^−3^), and AABW (28.2 to 28.3 kg m^−3^). The underbar denotes the dominant source of the isopycnal change in each layer. The rightmost column presents the five-section average, and the ratio of the frontal shift/residual term to the total isopycnal change is shown in brackets. For 112°E, the leastwise SB shift is shown due to its absence from the 1996 measurement.

**From 1996 to 2019**			**112°E**	**120°E**	**128°E**	**140°E**	**150°E**	**Average**
Poleward SB shift [km]		1.5°C	> 43	120	55	55	92	73
		Dynamic height	27 ± 2	74 ± 20	13 ± 4	49 ± 1	38 ± 14	40
Isobaric change	*d*Θ [°C]	Upper 1500 dbar	+0.10 ± 0.04	+0.10 ± 0.04	+0.10 ± 0.16	+0.32 ± 0.12	+0.30 ± 0.21	+0.18
		Lower 1500 dbar	+0.10 ± 0.02	+0.12 ± 0.03	+0.13 ± 0.03	+0.17 ± 0.06	+0.11 ± 0.06	+0.13
	$\begin{array}{c} {\mathrm{dS}}_{A}\\ \left[g\;{\text{kg}}^{-1}10^{-3}\right] \end{array}$	Upper 1500 dbar	+1 ± 4	+3 ± 4	+0 ± 19	+8 ± 12	+18 ± 17	+6
		Lower 1500 dbar	−6 ± 4	−3 ± 5	−0 ± 5	+1 ± 8	−5 ± 6	−3
	$\begin{array}{c} d\gamma_{n}\\ \left[\text{kg}\;m^{-3}10^{-3}\right] \end{array}$	Upper 1500 dbar	−16 ± 3	−13 ± 8	−17 ± 7	−37 ± 12	−21 ± 14	−21
		Lower 1500 dbar	−27 ± 4	−26 ± 4	−25 ± 4	−30 ± 6	−27 ± 11	–27
Isopycnal change	*d*Θ [°C]	Upper CDW	+0.23 ± 0.19	+0.42 ± 0.44	+0.20 ± 0.40	+0.23 ± 0.25	+0.49 ± 0.38	+0.31
		Lower CDW	−0.04 ± 0.02	−0.00 ± 0.04	−0.06 ± 0.11	+0.02 ± 0.03	+0.04 ± 0.07	−0.01
		AABW	−0.07 ± 0.01	−0.03 ± 0.02	−0.03 ± 0.02	−0.02 ± 0.03	−0.04 ± 0.04	−0.04
	Frontal shift	Upper CDW	+0.06 ± 0.08	+0.32 ± 0.45	+0.10 ± 0.07	+0.38 ± 0.33	+0.19 ± 0.47	+0.21 (0.7)
		Lower CDW	+0.01 ± 0.00	+0.05 ± 0.04	+0.05 ± 0.03	+0.08 ± 0.05	+0.07 ± 0.07	+0.05 (−5)
		AABW	+0.00 ± 0.00	+0.03 ± 0.03	+0.01 ± 0.01	+0.03 ± 0.03	+0.02 ± 0.02	+0.02 (−0.5)
	Residual	Upper CDW	+0.15 ± 0.11	+0.10 ± 0.44	+0.10 ± 0.40	−0.15 ± 0.40	+0.30 ± 0.60	+0.10 (0.3)
		Lower CDW	−0.05 ± 0.02	−0.06 ± 0.06	−0.11 ± 0.14	−0.05 ± 0.06	−0.03 ± 0.10	−0.06 (6)
		AABW	−0.07 ± 0.01	−0.06 ± 0.05	−0.03 ± 0.03	−0.05 ± 0.03	−0.06 ± 0.04	−0.05 (1.4)

To further quantify the effects of the displacement of the barotropic ACC fronts offshore, the dynamic height (geopotential) between 300 to 1800 dbar is calculated (see Materials and Methods). Air-sea interactions do not penetrate this layer because oxygen-depleted water presents at 300 dbar in the vicinity of the SB (Fig. 1B). A poleward shift of the dynamic height contour is observed in the five meridional sections as well as the 1.5°C isotherm (Fig. 2C). The SB generally corresponds to a dynamic height of 4.0 to 4.5 m^2^ s^−2^. Near the SB, the poleward shift of the dynamic height contour (40 km by the five-section average; Table 1) is comparable to but notably smaller than the displacement of the isotherm (73 km) by about 40%. Although baroclinic eddies may partially influence local dynamic height values, it is known that the dynamic height consistently reproduces the position of the barotropic ACC fronts north of the SB (*15*). The SB is a baroclinic water mass boundary, not a dynamical front, and hence is not necessarily accompanied by a barotropic front (*11*, *12*). Orsi *et al.* (*11*) indicated a spatial difference between the 1.5°C isotherm and the dynamic height contour in the Indian sector. Therefore, the significant disagreement between the displacements of the SB and the dynamic height contour can be explained by baroclinic changes. The isopycnal flux (due to eddies and baroclinic flows) can cause this by baroclinically transporting warm CDW poleward.

### Isopycnal changes due to frontal shifts and enhanced overturning circulation

**Fig. 3:**
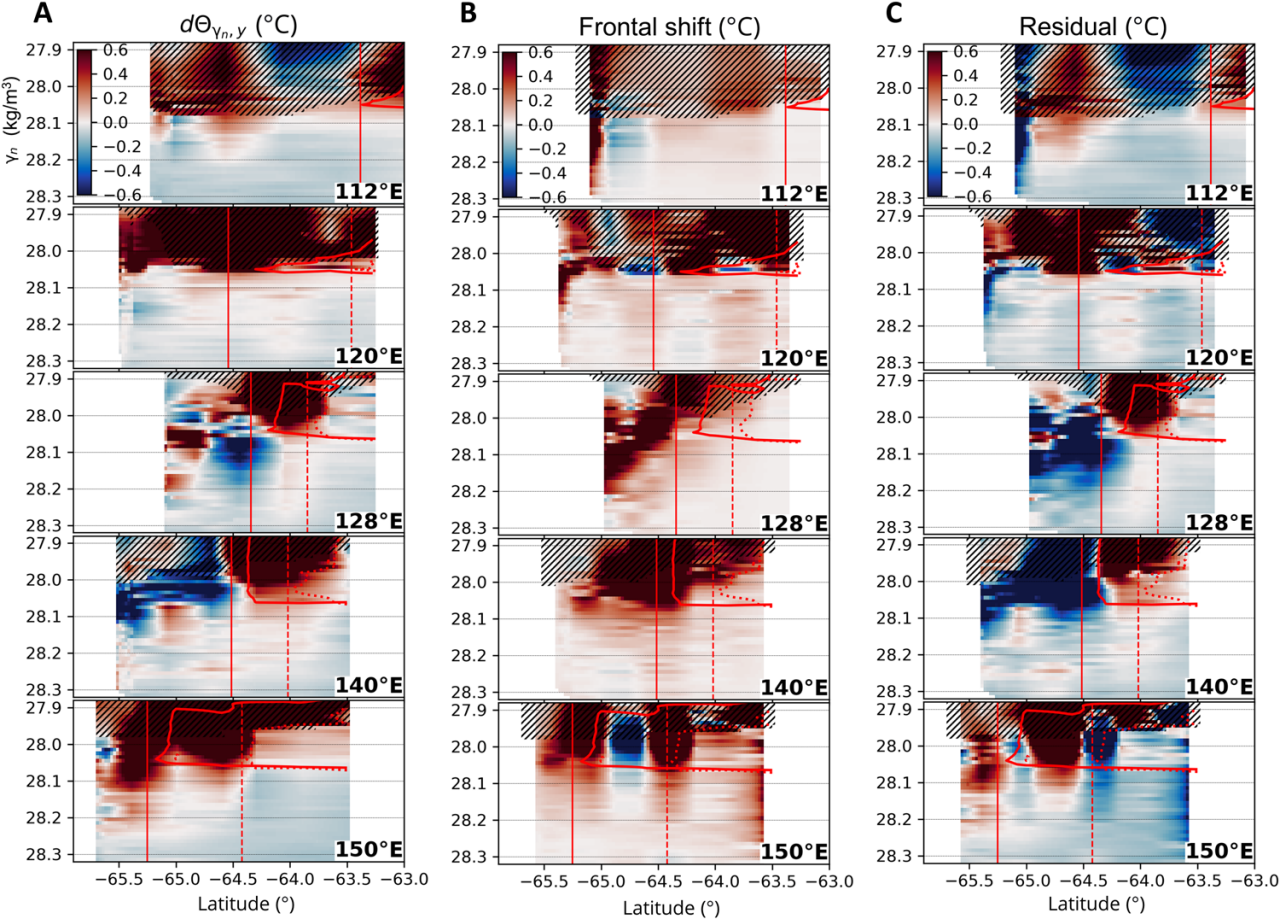
Isopycnal trends and frontal term decomposition. (**A**) Difference in the conservative temperature in the latitude-neutral density coordinates between 1996 and 2019. The 1.5°C contour and the SB position (derived from the isobaric field; Fig. 2A) for the 1996 and 2019 measurements are presented as in Fig. 2. Above 200 dbar, the data are hatched to hide transient surface features. The total isopycnal change (A) is decomposed into (**B**) the frontal shift and (**C**) residual (water transformation) terms. Upper CDW (28.0 to 28.1 kg m^−3^), Lower CDW (28.1 to 28.2 kg m^−3^), and AABW (28.2 to 28.3 kg m^−3^) are defined based on the extent of the temperature/salinity maximum layer (Fig. 1, C and D).

The poleward shift of the SB can be induced by both shifting the barotropic fronts and changing the water properties. The temperature trend is mapped onto the neutral density coordinates to eliminate transient features due to isopycnal heaving (Fig. 3A). The isopycnal analysis enables us to diagnose the cause of the meridional SB shift. Below the 28.0 kg m^−3^ isopycnal, the effect of air-sea interactions is negligible by the presence of oxygen-depleted water (Fig. 1B). Near the SB, the isopycnal temperature increases substantially in the Upper CDW layer (28.0 to 28.1 kg m^−3^) by about 0.31°C (by the five-section average; Table 1), but no significant increase is visible in the Lower CDW layer (28.1 to 28.2 kg m^−3^). The warming signal is generally confined above the 28.2 kg m^−3^ isopycnal (Fig. 3A), in contrast to the full-depth isobaric temperature increase (Fig. 2A). On isopycnals, a temperature change must be accompanied by a salinity change because seawater density at reference pressure is determined by temperature and salinity. Temperature decrease by 0.04°C in the AABW layer (28.2 to 28.3 kg m^−3^) is thus associated with salinity decrease by 0.007 g kg ^−1^, consistent with the previously reported freshening trend (*32*, *33*).

We decompose the isopycnal temperature change into two terms: the lateral movement of the water column and the along-streamline water transformation. The former results from the displacement of barotropic ACC fronts, whereas the latter results from baroclinic changes such as enhanced isopycnal fluxes (due to eddies and baroclinic flows), buoyancy forcing (e.g., freshwater inputs), and modified mixing. The effect of the barotropic frontal shift can be quantified by assuming that the dynamic height contours (Fig. 2C) accurately represent the ACC’s streamlines, which is supported by the gravest empirical mode methodology (*15*). On the basis of this assumption, the isopycnal warming due to the barotropic frontal shift (referred to as “frontal shift term”) is derived (see Materials and Methods). The frontal shift term (Fig. 3B) suggests that the poleward shift of the offshore ACC fronts generally explains the total temperature increase on isopycnals (0.21°C in Upper CDW and 0.05°C in Lower CDW; Table 1). At 64.8°S, 150°E, partial cooling occurs due to the eddy-like feature encountered in 1996 (*31*). The temperature increase due to the frontal shift term reaches the southern end of the meridional sections.

The along-streamline component of the isopycnal change (referred to as “water transformation term”) is obtained as the residual term. The water transformation term (Fig. 3C) indicates that temperature decreases associated with the widespread freshening of surface, bottom, and intermediate waters counteract the isopycnal warming caused by the poleward frontal shift (Fig. 3B) and the isopycnal flux. A substantial temperature increase exceeding 0.1°C due to the water transformation term is discernible at the poleward flank of Upper CDW (Fig. 3C; between the 1.5°C isotherms in 2019 and 1996). This along-streamline warming is not apparent to the north of the SB from the 1996 measurement. To the south of the SB, warming due to the water transformation term is observed at 112°, 120°, and 150°E, but not at 128° or 140°E. Because surface forcing is negligible below the oxygen-depleted layer (Fig. 1B) and the isopycnal flux likely overwhelms the diapycnal flux within the region of interest away from topographic boundaries, the most plausible explanation for the along-frontal warming is an intensification of isopycnal transport due to enhanced upper overturning circulation, which is likely driven by the poleward eddy flux (*7*, *10*). Because the mean flow is likely directed southward at 112°, 120°, and 150°E spatially associated with the southward excursion of the SB (*12*, *26*), the zonal difference to the south of the SB (Figs. 2 and 3) may be attributable to changes in the subpolar gyre. A relatively small temperature increase in the water transformation term (up to 0.1°C) is observed in a few sections (e.g., 150°E) below the 28.1 kg m^−3^ isopycnal, where the upper overturning circulation is unlikely to affect the temperature. This change may be related to a modified mixing ratio of the locally produced AABW (*22*, *32*) and salinification of the ambient Ross Sea Bottom Water (*34*). By the five-section average (Table 1), the warming of Upper CDW associated with the poleward shift of the SB (0.31°C) is about 70% (0.21°C) due to the frontal shift term and about 30% (0.10°C) due to the water transformation term.

### Spatial variations in the multidecadal trend

**Fig. 4:**
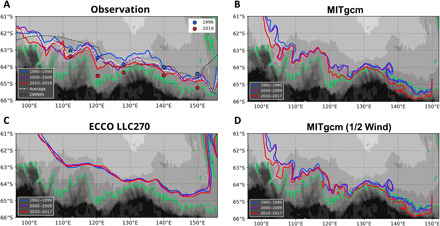
Regionality in the multidecadal trend. (**A**) SB position obtained from the 1996/2019 hydrographic measurements (dots) and the decadal climatology (curves; fig. S3). The triangle at 112°E for 1996 denotes the absence of the SB, assumed to exist northward of this position. The white dashed curve denotes the climatological position (three-decade average), and the dashed black curve is the SB from Orsi *et al.* (*11*). Background color indicates the bathymetry, and the 3000-m isobath is highlighted in green. (**B**) SB position reproduced from the high-resolution numerical simulations by MITgcm (nominally 1/30° grid). (**C**) Same as (B), but from the ECCO LLC270 ocean state estimate product (nominally 1/4° grid), which is used for the lateral boundary condition of (B). (**D**) Same as (B) but from the halved wind experiment of the high-resolution simulation.

We further investigate the multidecadal temperature trends using different datasets. Historical in situ measurements from the 1990s onward are compiled (fig. S1) to determine the decadal temperature variations at the subsurface temperature maximum (see Materials and Methods). The climatological SB position (defined by the 1.5°C isotherm) reproduced from the compiled datasets is generally consistent with previous estimates (Fig. 1A) (*11*, *12*, *35*). The decadal climatology further supports our finding that the SB has shifted poleward over the past three decades (Fig. 4A and fig. S2A). We estimate the overall poleward shift from 1990–1999 to 2010–2019 as 49 ± 25 km (between 100° and 150°E; see fig. S3 for the decadal temperature fields off East Antarctica). The SB shift accompanies a significant temperature increase by 0.1° to 0.6°C (95% confidence level) from 1990–1999 to 2000–2009 (fig. S3D) and by 0.1° to 0.4°C from 2000–2009 to 2010–2019 (fig. S3E). Therefore, the comparisons of the observations made in 1996 and 2019 (Figs. 2 and 3, and Table 1) very likely represent the multidecadal trend, rather than possible aliasing due to interannual variations.

The poleward displacement of the SB is the greatest at 120°E among the five meridional sections (120 km; Table 1). This regionality is consistent with the compiled historical data, which exhibit a maximum shift of ~80 km at this longitude (from 1990–1999 to 2010–2019; Fig. 4A and fig. S2A). The displacement of the SB and the temperature increase (Fig. 4A and fig. S3, D and E) are likely prominent between the topographic spurs in the continental slope (particularly at 105°, 110°, and 120°E), where the climatological SB deviates southward. The SB derived from pre-1990 data (*11*) is also presented in Fig. 4A (dashed black curve). Despite the limited number of synoptic sections at the time used to determine its position (generally 10° longitude intervals), poleward shifts from the pre-1990 data are visible at 105° to 120°E and east of 140°E. From the continental rise (3000 m) to the shelf break (1000 m) at 100° to 115°E and around 125°E, the temperature increases continuously for the three decades (fig. S3, D and E). The localized shifts and warming patterns are likely related to the cross-slope water exchange maintained by the clockwise subgyres bounded by the continental slope (*12*, *26*). The larger isotherm shifts in the CDW intruding locations may be caused by enhanced subpolar gyre transport.

Our numerical simulations with an eddy-resolving grid spacing (nominally 1/30°), laterally constrained by a simulation-based historical state estimate [Estimating the Circulation and Climate of the Ocean (ECCO); see Materials and Methods] (*36*), successfully reproduce the multidecadal poleward shift of the SB with a regionality similar to that of the compiled historical dataset (Fig. 4B and fig. S2B). An overall poleward shift of 43 ± 36 km occurs from 1992–1999 to 2010–2017 at 100° to 150°E, and the most prominent displacement of ~60 km occurs at 120°E. The SB is located southward of the 3000-m isobath east of 125°E and northward of 61°S west of 100°E, possibly because the simulated subpolar gyre is stronger than the reality. Despite this inevitable difference between the simulated and observed SB positions, the multidecadal poleward shift is reproduced over a wide range of longitudes, consistent with the observations (105° to 110°E and 120° to 140°E; fig. S2, A and B). The ECCO data used for the simulation (nominally 1/4° grid) generally reproduce the multidecadal warming of Upper CDW further north (*37*). However, the poleward shift of the SB is not evident as in the eddy-resolving experiment (Fig. 4, B and C). This result may be caused by differences in the model configuration or the grid spacing because mesoscale processes with a horizontal scale of ~10 km (*38*) are not resolved in the 1/4° grid. The barotropic transport of the simulated subpolar gyre significantly increases from 1992–1999 to 2010–2017 (between 115° and 125°E; fig. S4). This result is consistent with that of a circumpolar study, in which the intensified westerlies yielded enhanced gyre transport (*28*). Furthermore, halving the air-sea drag coefficient in the simulation (see Materials and Methods), which effectively halves the eastward wind stress over the ACC, qualitatively modifies the locality in the trend (Fig. 4D). The poleward shift at 120° to 125°E disappears, whereas the position of the SB between 110° and 120°E changes from along-slope to cross-slope and becomes temporally transient. This experiment suggests that the multidecadal trend is partially driven by regional wind forcing, which likely alters shoreward eddy transport (*7*, *14*) and the strength of the barotropic flow (*39*, *40*).

## DISCUSSION

On the basis of in situ hydrographic measurements and numerical eddy-resolving simulations, we found evidence for a multidecadal poleward shift of the SB off East Antarctica. This study sheds light on the mechanism of multidecadal warming of the CDW layer in the subpolar zone (*1*–*5*) and extends our knowledge of the multidecadal variability of the ACC in a changing climate.

The comparisons between the 1996 and 2019 measurements suggest that the poleward shift of the SB is driven by the poleward shift of barotropic ACC fronts (Fig. 3B) and enhanced upper overturning circulation due to increased isopycnal fluxes by eddies and/or baroclinic flows (Fig. 3C). The enhanced isopycnal fluxes associated with the upper overturning can compensate for the subsurface volume removal due to the anomalous northward transport at the surface driven by the intensified westerlies over the past decades (*6*, *17*). Because the Upper CDW temperature change governs the position of the SB, its poleward shift (accounting for +0.31°C; Table 1) can be attributed to enhanced upper overturning and the poleward shift of offshore barotropic fronts, generally accounting for 30% (+0.10°C) and 70% (+0.21°C), respectively. Thus, the effect of the barotropic frontal shift is likely no less than the baroclinic water transformation. This result is supported by the displacement of dynamic height contour (40 km) that explains more than half of the observed SB shift (73 km). The significance of barotropic changes may manifest as unsaturated eddy conditions in the subpolar zone, sometimes referred to as “southern mode,” in which the barotropic response to wind forcing is likely more sensitive than in the ACC (*39*, *40*).

Uncertainty in the frontal term decomposition likely arises from an assumption that the displacement of the dynamic height contours represents the frontal shift of the ACC because baroclinic eddies can also affect the water column density. Previous studies have reported that temperature and salinity fields in the ACC constructed by the gravest empirical mode with a dynamic height of upper 2000 dbar capture over 90% of the property variance below the thermocline (*15*), and that the quasi-barotropic flow structure of the study region is well represented by dynamic height (*12*, *26*) as in the north. These facts assure that dynamic height approximates the position of barotropic ACC fronts in the presented result.

Even without the isopycnal analysis, a contribution from the poleward shift of barotropic fronts is evident. Neither enhanced eddy transport, altered mixing ratio, nor the freshening-driven volumetric contraction of AABW can explain the isobaric temperature increase with the deepening of the isopycnals throughout the water column (Fig. 2A) observed in this and previous studies (*22*, *33*). Although the circumpolar-averaged ACC position has not shifted over the past decades (*7*), the regional shift of the ACC fronts represented by the dynamic height (Fig. 2C) and detectable by satellite altimetry (*23*) has contributed significantly to full-depth warming in the subpolar zone. In contrast, we can expect that enhanced upper overturning caused the baroclinic poleward shift of the isotherms because this pertains to the discrepancy between the shifts in SB and dynamic height (Table 1). The isopycnal eddy flux likely increases in response to wind intensification (*13*, *14*). In the presented results, enhancement of eddy flux does not necessarily occur locally. Baroclinic changes can be transferred from upstream where active eddy generation is observed, such as the eastern flank of the Kerguelen Plateau (*20*, *41*). Enhanced isopycnal CDW upwelling in the upper overturning cell is consistent with the widely observed shoaling of the subsurface temperature maximum (*2*, *3*). Because the poleward shift of the barotropic fronts likely deepens the isopycnals, enhanced isopycnal transport can reasonably reconcile the shoaling of CDW (*41*).

The zonal variation in the poleward SB shift obtained from the 1996 and 2019 observations (Figs. 2 and 3) is consistent with that reproduced from the historical dataset (Fig. 4A), indicating that the SB shifted further poleward around 120°E associated with the continental slope topography. The eddy-resolving simulation reproduced the similar locality (Fig. 4B), supporting the topographic control on the poleward SB shift. Our simulation implicates the effect of mesoscale processes in the observed trend, which are characterized by the baroclinic Rossby radius (~10 km at the shelf break) (*38*) and are unresolved in the ECCO model (Fig. 4C). The configurations of the simulation (e.g., atmospheric forcing and eddy parameterization scheme), however, are not the same as the ECCO model (differences in the zonal wind stress between the models are unlikely to explain the absence of SB shift in the ECCO). The effects of mesoscale processes need to be scrutinized further. The locality in the multidecadal trend is likely related to a topographically controlled flow structure (*12*, *26*). The sea depth near the SB shallows by ~250 m over the three decades (averaged between 100° to 150°E), whereas the depth change at 120°E is the smallest (~150 m). Because the barotropic flow tends to follow the isobath to conserve potential vorticity, the furthest poleward shift at 120°E (Fig. 4) is likely explained by the flat bathymetry and the southward excursion of the continental slope at this longitude. The localized shifts correlated with the shoreward CDW intrusions (Fig. 4A and fig. S3), the zonally asymmetric baroclinic warming (Fig. 3C), and the barotropic transport increase (fig. S4) support the idea that the subpolar gyre strengthened over the period. This idea is consistent with implications from observations in the Weddell Sea (*5*), idealized models (*21*), and realistic circumpolar simulations (*28*).

The sensitivity experiment demonstrates the role of eastward wind stress over the domain (Fig. 4, B and D), which likely enhances the poleward eddy flux and the wind-driven upper overturning. Because the manipulation halves the air-sea drag coefficient and the effect of wind intensification from the 1990s onward (*18*), the smaller shift compared to the reference experiment (fig. S2B) can be interpreted as being due to a decreased wind effect. Although the westerlies do not strengthen from the 1990s to the 2000s on a decadal mean basis (*13*), the historical data indicate persistent warming and the poleward SB shift during this period (Fig. 4A and fig. S3D). The warming during the 2000s may have been induced by the cyclonic wind stress curl anomaly around the Antarctic Divergence, which likely enhanced CDW upwelling towards the Sabrina Coast (120°E) and accelerated land ice discharge (*42*). The linkage between the localized SB shift off the Sabrina Coast favored by topography and this on-shelf warming event requires further investigation.

The poleward shift of the SB under intensified westerlies potentially enhances onshore heat intrusion, leading to multidecadal warming of Antarctic shelf water (*3*). The extensive poleward shift of the SB (Fig. 4A) may result from the multidecadal wind intensification, while it may also be induced by the intrinsic variability of the ACC (*19*). The dominant intrinsic variability of the Southern Ocean is a multidecadal mode of the Weddell Gyre in the Atlantic sector with a 40- to 50-year duration (*43*). The eddy activity relative to the intrinsic variability in the Indo-Pacific sector is more dependent on external wind forcing than the Atlantic sector (*19*, *44*). In contrast to the Weddell and Ross Gyres, a synoptic-scale subpolar gyre is absent off East Antarctica (*12*, *26*). These facts imply a minor effect of the intrinsic variability within the domain and the sensitivity of the circulation to the projected wind intensification due to anthropogenic forcing (*6*, *7*, *45*). Isopycnal barriers of the Antarctic Slope Current are partially weak in the Indian sector (*45*), which may allow immediate onshore heat access in response to the offshore poleward SB shift. The presented result indicates the localized warming towards the shelf break (fig. S3, D and E), which is likely consistent with the continental shelf warming (*3*). Our findings remark the importance of sustained observations of the ocean interior off East Antarctica.

## MATERIALS AND METHODS

### Hydrographic data

High-quality hydrographic observations in the Australian-Antarctic Basin were obtained during the austral summers of 1996 [R/V *Aurora-Australis*: Expocode 09AR9604_1; (*31*)] and 2019 [R/V *Kaiyo-maru*: Expocode 490S20181205 and 490S20190121; documented in (*34*)] and were compared. The year in the text denotes the new year of the season (i.e., cruises from December 2018 to March 2019 are denoted as 2019). Both hydrographic datasets are available through the CLIVAR (Climate and Ocean: Variability, Predictability and Change) and Carbon Hydrographic Data Office website (https://cchdo.ucsd.edu/). The position of the SB was determined in each meridional section as the southernmost extent of the 1.5°C isotherm. Temperature values of the temperature maximum surface at each station were linearly interpolated to obtain the meridional temperature profile. The poleward limit of the 1.5°C isotherm was then determined from the interpolated profile. For the 1996 observations, the SB at 150°E did not correspond to the southernmost isotherm isolated by the cold eddy at 64.6°S. This choice of SB is supported by the robust isobaric/isopycnal temperature increases from 1996 to 2019 (Figs. 2A and 3A) and the smoothed meridional profile of the dynamic height (Fig. 2C) along this section. The accuracies of the temperature and salinity data from the two measurements are ~0.002°C and ~0.003 g kg^−1^, respectively.

### Isopycnal analysis

To determine the cause of the isopycnal water property changes, the following derivative rule was applied for the isopycnal temperature change

$$d\Theta_{y}=d\Theta_{\psi }+{\frac{d\Theta }{d\psi }|}_{y}d\psi_{y}$$ (1)

where Θ, *y*, and ψ are the Conservative Temperature, latitude, and dynamic height, respectively, and the subscript denotes manipulation by fixing the variable. The left-hand side of Eq. 1 is the Eulerian change on the latitude coordinates, simply the observed isopycnal change. The first term on the right-hand side corresponds to the along-streamline water transformation term, i.e., the temperature change along the geostrophic contour of the barotropic ACC front ψ. The second term is the temperature change due to the meridional shift of ψ, referred to as the frontal shift term. The frontal shift term was explicitly calculated, whereas the water transformation term was derived as the residual of Eq. 1. The dynamic height at 300 to 1800 dbar was adopted as the proxy for ψ based on the fact that the dynamic height above 2000 dbar accurately represents the position of the barotropic ACC fronts (*12*). The dynamic height was calculated by vertically integrating the specific volume (inverse potential density anomaly). The presented results are not sensitive to the choice of depth range, while the temperature above 300 dbar may have been more influenced by short-lived surface features than by the frontal position. To estimate the dynamic height change on the latitude coordinates, the meridional profile of the dynamic height was created by fitting the dynamic height value at each station with a quartic polynomial curve (Fig. 2C). This fitting smoothed out eddy-like features that are irrelevant to the frontal position (e.g., the cold eddy at 150°E during the 1996 observations) and enabled us to obtain dynamic height profiles monotonically increasing northward. Using the meridional dynamic height profiles, the mean isopycnal temperature gradients on the ψ coordinates were calculated, and subsequently, the frontal shift term was derived.

### Historical data analysis

A dataset of conservative temperature on the temperature maximum surface (which characterizes Upper CDW) was constructed from the historical data from 1990 to 2019 (figs. S1 and S3). This dataset comprises shipboard conductivity-temperature-depth (CTD), expendable CTD (XCTD), Argo float, and biologging profiles. The shipboard CTD, XCTD, and float data used in the analysis are available through the National Centers for Environmental Information (https://ncei.noaa.gov/) and the Japanese Institute of Cetacean Research (https://icrwhale.org/) websites, and the biologging data are available through the Marine Mammals Exploring the Oceans Pole to Pole website (http://meop.net/). First, the temperature profiles were vertically interpolated every 1 dbar using Akima interpolation. The targeted temperature was extracted as the temperature maximum in the interpolated vertical profile below the temperature minimum above 500 dbar. When calculating the conservative temperature, a climatological salinity field from the gridded climatological dataset (*35*) was used. The consistency of the climatological position of the SB reproduced from this climatological product supports its fidelity (Fig. 1A and fig. S3). The error associated with this treatment of salinity data has only a minor effect on the conservative temperature relative to the measurement error of the dataset (accurate to 0.01°C). The errors in the pressure/depth measurements are also negligible for the conservative temperature values at the detected temperature maximum surface. Objective mapping of the sparsely distributed surface data onto 1/4° grids was performed using radial basis function interpolation with a linear weighting function, which can smoothly interpolate noisy data in a least-squares sense (*12*). Areas with less than three data points within a radius of 150 km from the grid point were masked (figs. S1 and S3). The SD of the meridional position of the SB (fig. S2A) was derived as follows: The interpolated absolute deviation (residuals) of the temperature maximum surface was first calculated as the root mean square residual from the objectively mapped surface of the climatological dataset. Second, the mean temperature gradient of the objectively interpolated field was calculated to derive the SD of the horizontal displacement of the SB. By multiplying this by the meridional factor of the mean temperature gradient vector at each location, we obtained the SD of the meridional position of the SB as 56 ± 10 km between 100° and 150°E (fig. S2A). This analysis enabled us to evaluate the significance of the multidecadal poleward shift of the SB recorded in the historical data. To determine the statistic applicable for Welch’s *t* test of the objectively interpolated fields (fig. S3, D and E), we made two assumptions: (i) The interpolated absolute deviation closely approximates the SD field, and (ii) the sample size in each grid is given by the number of surface data points within a radius of 150 km from the grid point.

### Numerical simulations

High-resolution numerical simulations were conducted using the Massachusetts Institute of Technology general circulation model (MITgcm; http://mitgcm.org/) and compared with the observed changes (Fig. 4, B and D). The model grid size was set to nominally 1/30° (3 to 4 km) with 50 vertical levels. The simulation was configured with hydrostatic approximations, dynamic/thermodynamic sea ice, and a thermodynamic ice shelf. The model domain covers 90° to 150°E and 60° to 70°S, spanning the period from 1992 to 2017. The bathymetry was prescribed by ETOPO1 (https://ngdc.noaa.gov/mgg/), with updates from recent hydrography over the continental shelves. Because the bathymetry in the target region is consistent among the different datasets, the choice of bathymetric data was unlikely to have affected our results. We adopted the ECCO estimate (https://ecco-group.org/), the multidecadal ocean state estimate powered by MITgcm, as the lateral boundary condition. LLC270 optimization data (nominally 1/4° grid and 50 vertical levels) were used (*36*). The LLC270 data were also analyzed to evaluate the simulation’s sensitivity to the grid size (Fig. 4C). Atmospheric forcing is based on the ERA-Interim reanalysis data (https://ecmwf.int/). Initial conditions were derived from a 25-year spin-up from an initial resting state. As we did for the in situ historical data, the output was vertically interpolated every 1 dbar using Akima interpolation to precisely derive the temperature maximum surface. The halved wind experiment (Fig. 4D) was performed by setting the overall scaling of the neutral drag coefficient (“exf_scal_BulkCdn”) to 0.5, which halved the air-sea interfacial stress.

## SUPPLEMENTARY MATERIALS

Supplementary material for this article is available at http://advances.sciencemag.org/cgi/content/full/7/24/eabf8755/DC1
